# Anxiety as a risk factor for cardiovascular disease independent of depression: A narrative review of current status and conflicting findings

**DOI:** 10.1177/2055102920987462

**Published:** 2021-01-13

**Authors:** Håvard R Karlsen, Florian Matejschek, Ingvild Saksvik-Lehouillier, Eva Langvik

**Affiliations:** 1 Norwegian University of Science and Technology, Norway; 2 University of Vienna, Austria

**Keywords:** anxiety, cardiovascular disease, coronary disease, review, stroke

## Abstract

The aim of this paper is to summarise and evaluate the empirical support for the association between anxiety and cardiovascular disease (CVD) and to address challenges related to method and study design. We review results from meta-analyses and more recent findings on the association of anxiety and the risk of CVD. Depression and anxiety are often listed as psychosocial risk markers of CVD, but the role of anxiety as a risk factor for CVD has not received the same evidential support as the effects of depression. Through a narrative review we identified six meta-analyses as well as 15 recent large studies of anxiety and CVD that we summarise. Some of the conflicting findings may be artefacts of study design or population the sample is drawn from. Researchers should take care to be population specific, measurement specific and outcome specific, and to control for comorbid depression.

## Introduction

Anxiety disorders are the most prevalent group of psychiatric disorders worldwide (Pérez-Piñar et al., 2016) with a reported lifetime prevalence as high as almost 29% (Kessler et al., 2005). Cardiovascular diseases (CVD), especially Coronary Heart Disease (CHD), are the leading cause of death in Europe (Townsend et al., 2015), as well as in China (Zhou et al., 2016). Considering the massive impact of both anxiety disorders and CVD in terms of mortality and quality of life, further enquiry into a possible association between them appears both relevant and necessary. While research has mainly focused on depression, which is an obvious major psychiatric ailment, and has identified it as an independent risk factor for the development of CVD (Lichtman et al., 2014; Pan et al., 2011), the research on anxiety’s association with CVD has not yielded the same conclusive results so far. The aim of this narrative review article is to summarise recent findings and challenges in the research field.

## Methodology

We searched online for papers that examined the relationship between anxiety and CVD while controlling for the potential confounding effects of depression. Databases we searched were MEDLINE, Psychinfo, Global Health and Google Scholar, using these keywords: ‘anxiety or anxiety disorder or generalised anxiety disorder or panic or panic disorder and cardiovascular disease or heart disease or heart attack or myocardial infarction or stroke’. We excluded papers that did not control for depression. While we mainly focus on general anxiety disorder and anxiety in general, we also discuss panic disorder briefly. We primarily included longitudinal studies published since 2009.

## Controlling for depression in studies of anxiety and CVD outcomes

In a meta-analysis conducted by Roest et al. (2010), the presence of an anxiety disorder was found to increase the risk for both incident CHD (HR = 1.26; 95% CI: 1.15–1.38) and cardiac mortality (HR = 1.48; 95% CI: 1.14–1.92). While these results imply an association between anxiety and a subclass of CVD, namely CHD, it must be noted that only a very small amount of the studies included in this meta-analysis controlled for depression. This is problematic since the two disorders often co-occur and show similar symptoms that can be difficult to differentiate (Jacobson and Newman, 2017). Results comparable to the aforementioned meta-analysis have been reported by Emdin et al. (2016), although it should be noted that the latter study also lacks the inclusion of depression as a control. An indication of how vital it is to account for depression is evident in the meta-analysis by Celano et al. (2015), which included 32 studies, of which only 13 controlled for depression. While the authors did find a significant non-adjusted association of anxiety and mortality in patients with CHD, the role of depression might have attenuated this relation. When they included only the 13 studies accounting for depression as a covariate in a sensitivity analysis, they found no remaining significant association between anxiety and mortality. Tully et al. (2015) found an increased risk of CHD in people with panic disorder, a sub-diagnosis of anxiety in their meta- analysis. This effect persisted in studies that excluded cases of depression and in studies that adjusted for depression. Two meta-analyses have examined the relationship between anxiety and cerebrovascular disease (CER; Batelaan et al., 2016; Pérez-Piñar et al., 2017). Both identified a significant increase in risk of CER, though neither controlled for the effect of depression. Key characteristics of the discussed meta-analyses are presented in Table 1. Recent research has increasingly taken into consideration the importance of accounting for co-occurring depression when investigating the link between anxiety and CVD. For example, a meta-analysis by Batelaan et al. (2016) including 14 studies controlling for or removing cases of depression reports an association between anxiety and an increased risk for incident CVD (HR = 1.57, 95% CI 1.29–1.90). In a large retrospective cohort study by Liu et al. (2019) including 32,345 US-participants initially free of CHD, a significant association between Generalised Anxiety Disorder (GAD) and CHD was found (RR = 2.09; 95% CI: 1.22–3.58). A prognostic cohort study also conducted in the USA amongst 2041 initially CVD-free primary care patients yielded similar results: Patients who screened positive for anxiety at baseline had an elevated risk of a CVD event up to 3 years after baseline evaluation (Stewart et al., 2016). As this sample consisted predominantly of older and socioeconomically disadvantaged individuals, it remains unclear if the findings of Stewart and colleagues can be applied to the general US-population. The authors, however, stress the importance of the inclusion of usually under-represented groups.

**Table 1.** Key characteristics of discussed meta-analyses.

Study	Number of included studies	*N*	Results (95% CI)
Batelaan et al. (2016)	37	1,565,699	CVD: HR = 1.52 [1.36, 1.71]; Only studies adjusting for depression: HR = 1.57 [1.29, 1.90]
Celano et al. (2015)	44	30,527	Dic. Anxiety measure: Mortality: OR = 1.30 [0.98, 1.73]; Composite outcome: OR = 1.20 [0.91, 1.58]; Cont. Anxiety measure: Mortality: OR = 1.08 [0.90, 1.30]; Composite outcome: 1.21 [1.05, 1.39]
Emdin et al. (2016)	46	2,017,126	CV mortality: RR = 1.41 [1.13, 1.76]; CHD: RR = 1.41 [1.23, 1.61]; stroke: RR = 1.71 [1.18, 2.50]; HF: RR = 1.35 [1.11, 1.64]
Pérez-Piñar et al. (2017)	8	950,759	Stroke: HR = 1.24 [1.09, 1.41]
Roest et al. (2010)	20	249,846	CHD: HR = 1.26 [1.15, 1.38]; Cardiac mortality: HR = 1.48 [1.14, 1.92]
Tully et al. (2015)	12	1,131,612	CHD, panic disorder: adjusted HR = 1.47 [1.24, 1.74]. Excluding depression cases: adjusted HR = 1.64 [1.45, 1.85]

CVD: Cardiovascular disease; HR: Hazard ratio; Dic.: dichotomous; OR: Odds ratio; Cont.: continuous; CV: Cardiovascular; CHD: Coronary Heart disease; RR: Relative Risk; HF: Heart Failure.

Some studies, on the other hand, report no significant association between anxiety and CVD in initially CVD-free cohorts. In a prognostic cohort study including 853 Greek adults, Kyrou et al. (2017) reported an elevated adjusted risk of a CVD event for depression (OR = 3.6, 95% CI: 1.3–11) while there was no stable effect of anxiety (OR = 1.03, 95% CI: 1.0–1.1). In a study of 3135 elderly American men, anxiety was unrelated to either CHD or cerebrovascular disease (Karlsen et al., 2020). The analyses were adjusted for the effect of depression, and there was no effect of anxiety in either the group with a prior history of CVD or the group with no prior history.

## Prognostic approaches to anxiety and CVD

A number of studies have applied a prognostic approach, i.e. focused on the association between anxiety and CVD in individuals who have previously experienced CVD events in their lifetime. These studies present similarly heterogeneous results as the CVD-free cohorts described above, not least owing to the high variety in sample characteristics. One of them, a study (AbuRuz et al., 2018) investigating the association of anxiety with Acute Myocardial Infarction (AMI) in Jordanian CHD-patients, reports a significantly elevated risk of an AMI-event for anxious CHD-patients (OR = 1.55; 95% CI: 1.15–2.10). However, several studies did not find such an association in other post-CVD samples with a more general CVD outcome. Nakamura et al. (2013) observed a significant association of depression, but not anxiety, with cardiovascular hospitalisation or death. Further, in a Danish cohort of 610 CHD patients, Versteeg et al. (2013) did not find a significant association between anxiety and cardiovascular hospitalisation or death, while depression was independently associated with both outcomes.

Adding to the complexity, study populations have included patients suffering from a variety of different diseases at baseline. Bruce et al. (2016) report an elevated risk of cardiovascular mortality, but not of incident CHD, for type 2 diabetes patients with GAD. In a Spanish study, anxiety was not significantly associated with an adverse cardiovascular event or mortality in a sample with metabolic syndrome (Ortega et al., 2018). Surveying a sample of female breast cancer survivors free of CVD in the Netherlands, Schoormans et al. (2017) found a significant association of pharmaceutically treated anxiety and CVD.

## Conflicting findings

Some studies have found increased CVD risks from certain sub-diagnosis of anxiety, but not from others. Studies that have included generalised anxiety disorder as well as other sub-diagnosis of anxiety have found increased CVD risks of panic disorder, but not of generalised anxiety disorder (Seldenrijk et al., 2015; Tully and Baune, 2014). Aside from being addressed as a potential risk factor, anxiety has even been suggested as a cardio-protective factor in the context of CVD. Langvik and Nordahl (2014) found that anxiety reduced the risk of AMI in a large, longitudinal population survey, when controlling for depression. In a cross-sectional study by Huang et al. (2009) on the population of Taiwan, participants with an anxiety disorder, but no depression had a higher risk of having comorbid CHD or hypertension compared to healthy controls. The risk was greater for the younger age groups (<45 years) and reversed for those older than 64 years. Hence, older participants with anxiety were less at risk of having CHD or hypertension than healthy controls in the same age-group. In a study by Parker et al. (2011), the presence of Generalised Anxiety Disorder (GAD) in patients with acute coronary syndrome (ACS) significantly improved cardiac outcome, defined as a hard CVD event (for baseline GAD: OR = 0.35; 95% CI: 0.17–0.75; for lifetime GAD: OR = 0.42; 95% CI: 0.23–0.78). Key characteristics of the aforementioned single studies can be found in Table 2. This effect was, however, limited to patients suffering from GAD only and did not appear in conjunction with other anxiety disorders. A possible explanation offered by the authors is that GAD-patients might be more likely to seek medical assistance when experiencing somatic symptoms possibly stemming from their previous cardiac event. Additionally, greater adherence to therapy options and professional advice are also listed as plausible explanations (cf. Benyamini et al., 2013).

**Table 2.** Key characteristics of discussed single-studies.

Study	*N*	Sample (country)	Mean age (SD/range)
AbuRuz et al. (2018)	1000	CHD patients (Jordan)	66.6 (11.1)
Bruce et al. (2016)	1337	Type 2 diabetes (Australia)	64.9 (14.4)
Huang et al. (2009)	1,031,557	Whole population (Taiwan)	Four groups: <20, 20–44, 45–64, 65⩽. No information on distribution
Karlsen et al. (2020)	3095	Community sample (US)	76.4 (5.5)
Kyrou et al. (2017)	853	CVD-free (Greece)	F: 44 (18), M: 45 (13)
Langvik and Nordahl (2014)	41,248	CHD-free (Norway)	Non-MI: F: 43.12 (13.07), M: 43.61 (12.90), MI: F: 57.87 (9.31), M: 55.81 (9.44)
Liu et al. (2019)	32,345	CHD-free (US)	45.3 (17.2)
Nakamura et al. (2013)	414	CVD (Japan)	64.9 (13.1)
Ortega et al. (2018)	401,743	MetS (Spain)	60.11 (9.9)
Parker et al. (2011)	489	ACS (Australia)	65.7 (12.2)
Schoormans et al. (2017)	7227	CVD-free breast cancer survivors (Netherlands)	CVD: 70 (46–91); No CVD: 60 (23–102)
Seldenrijk et al. (2015)	2510	CVD-free (Netherlands)	41.2 (18–65)
Stewart et al. (2016)	2041	CVD-free primary care patients (US)	68.5 (6.9)
Tully and Baune (2014)	4181	Stratified sample (Germany)	43.5 (SD 11.6, range 18–65)
Versteeg et al. (2013)	610	CHD-patients (Denmark)	65.8 (10.8)

Sex	Follow-up (years)	Anxiety type (measure)	Outcome	Results (95% CI)
M + F	2	Anxiety (HADS)	MI	OR = 1.55 [1.15, 2.10]
M + F	4	GAD (GADS)	All-cause mortality + CV-mortality + incident CHD	CVMort: HR = 4.60 [1.62, 13.08], CHD: HR = 1.26 [0.67, 2.36]
M + F	0 (cross-sectional)	Anxiety disorders (diagnosis)	CHD	Average RR for age groups: <20 = 9.88, 20–44 = 3.86, 45–64 = 1.4, 65⩽ = 0.66
M	12	GAD (GADS)	CHD + CER	CHD HR = 0.95 [0.71, 1.27], CER HR = 1.33 [0.69, 1.77]
M + F	10	Anxiety (STAI-state anxiety subscale)	CVD	OR = 1.03 [1.0, 1.1]
M + F	7.2	Anxiety (HADS)	MI	OR = 0.61 [0.50, 0.79]
M +	F 3	Anxiety disorders (AUDADIS-IV + psychiatric diagnoses)	Incident CHD	RR = 2.09 [1.22, 3.58]
M + F	1.18	Anxiety disorders (GAD-7)	CV-hospitalisation or death	HR = 2.35 [0.77, 6.18]
M + F	4.91	Anxiety disorder diagnosis (Health databank)	Incident CV-events + mortality	RR = 0.99 [0.95, 1.02]
M + F	5	Anxiety disorders (CIDI + research assistant’s verdict + HADS-A)	Cardiac admission/death/event	GAD: 5-year OR = 0.35 [0.17, 0.75], lifetime OR = 0.42 [0.23, 0.78]
F	13	Anxiety (Drug dispenses for anxiety disorders)	CVD (at least two drug dispenses)	HR = 1.48 [1.05, 2.08]
M + F	5.5	Generalised anxiety disorders, panic disorder (DSM-IV, CIDI)	CVD	GAD: adjusted RR = 1.28 [0.71, 2.30], Panic disorder: adjusted RR = 2.12 [1.27, 3.55]
M + F	8	Anxiety (Prime-MD)	Hard CVD event (fatal/acute MI, stroke)	HR = 1.53 [1.20, 1.95] within 0–3 years of follow-up
M + F	0 (12-month prevalence)	Generalised anxiety disorders, panic disorder (DSM-IV, CIDI)	CVD	GAD: adjusted OR = 0.94 [0.37, 2.37], Panic disorder: adjusted OR = 2.89 [1.47, 5.69]
M + F	5	Anxiety (HADS)	Cardiac-related hospitalisation or all-cause mortality	HR = 0.96 [0.70, 1.32] for first hospitalisation

CHD: coronary heart disease; HADS: Hospital Anxiety and Depression Scale; MI: myocardial infarction; GAD: generalised anxiety disorder; GADS: Generalised Anxiety Disorder Scale; CV: cardiovascular; STAI: state-trait anxiety inventory; AUDADIS: the alcohol use disorder and associated disabilities interview schedule; GAD-7: Generalised Anxiety Disorder 7-item Scale; MetS: metabolic syndrome; ACS: acute coronary syndrome; CIDI: composite international diagnostic interview; Prime-MD: primary care evaluation of mental disorders.

## Possible underlying pathways

With regards to the possible mechanisms linking anxiety to increased CVD-risk or worse CVD-outcomes in CV-patients, there are two main suggested pathways: A behavioural pathway and a biological pathway (Cohen et al., 2015; Pan et al., 2017).

On the behavioural level, quite similarly to depression, anxious individuals may adhere to poorer health behaviour, which subsequently increases their CVD-risk (Cohen et al., 2015). Examples of such behaviour are lower physical activity, cigarette smoking, excessive alcohol consumption and poor diet. While non-adherence to medication is an example of poor health behaviour well documented for depression (Benyamini et al., 2013; DiMatteo et al., 2000), its occurrence in anxiety seems to be a matter of debate (cf. Cohen et al., 2015).

From a biological perspective, anxiety, like other negative emotions and chronic stress, is assumed to alter autonomic nervous system function via excessive activation of the hypothalamic-pituitary-adrenal axis and the sympathetic nervous system (Cohen et al., 2015). This, in turn, causes endothelial damage due to an increased release of plasma catecholamines, which ultimately leads to the development of CVD, such as atherosclerosis, CAD and acute coronary events. The understanding of these mechanisms has been expanded on in recent years by evidence linking atherosclerosis to chronic inflammation, and not, as was the previous consensus, to a mere accumulation of cholesterol (Fioranelli et al., 2018). While an association has been established between depression and inflammatory markers (Kop et al., 2010), the relation between anxiety and inflammation is still inconclusive (Celano et al., 2018). In the case of a more concrete definition of an anxiety disorder however, namely GAD, results seem to indicate an association with inflammation markers in CHD-patients (Bankier et al., 2008).

## Discussion

Research addressing anxiety as a risk factor for CVD often presents itself as a challenging mosaic of varying definitions, measures and sample characteristics. This is to be expected, as the term CVD implies a very broad range of diseases and definitions. However, as the differing practices observed in many studies pose a hindrance to further understanding of a potentially very relevant association, we make several suggestions that are aimed at helping to determine the real association between anxiety and CVD:

Firstly, there exists considerable variety regarding sample characteristics, with some samples consisting of participants free of CVD, while the majority of studies investigates either CVD-samples or those with risk factors for CVD. More research on initially CVD-free samples representing the general population would make the interpretation of research results and the drawing of valid conclusions easier.

We have stated above the importance of any research on the association of anxiety with CVD to bear in mind the role of comorbid depression. While newly published studies do seem to control for depression more frequently, more studies should take this factor into consideration. It will be interesting to see if and how pooled results of meta-analyses change once more studies account for depression.

Furthermore, there is a lack of specificity in terms of measures utilised by researchers. While many authors choose to use screening measures for anxiety, the variance in screening questionnaires (see Table 2) often leads to quite different rates of detected anxiety across studies. Moreover, the use of cut-off criteria is often opaque, that is, it is unclear whether anxious participants are compared to an anxiety-free, or merely a lower-scoring, group. Davidson et al. (2005) discuss some of these challenges in relation to depression, and it is likely that these arguments are applicable to anxiety as well. We therefore suggest to either use clinical diagnoses in order to categorise participants into groups with or without a defined anxiety disorder, or to employ a valid screening measure and use its continuous anxiety scale or a cut-off that differentiates solid cases of anxiety from cases of no anxiety. Similarly, studies are not always specific in their measurement of the construct anxiety. As different sub-diagnoses of anxiety (GAD, panic disorder, phobias) can have a different impact on CVD risk, this should be considered by researchers. In the same vein, while some studies examine CHD or CER, or AMI and stroke specifically, others examine the broader category of CVD in general. A lack of specificity may obscure potential relationships that exist at the sub-categories of CVD. Although some researchers (cf. Batelaan et al., 2016) found that the effect of anxiety was not different across CVD subcategories, we would still recommend that researchers run separate analyses for CHD and CER outcomes.

## Limitations

A narrative review like this study falls short in comparison with a systematic review that would have increased the likelihood of including all relevant new findings. Narrative reviews are criticised for lacking the synthesis and rigour of a systematic review, but have the advantage of being broader in scope than systematic reviews (Byrne, 2016). Likewise, it would have been beneficial to follow the PRISMA checklist (Moher et al., 2009), to comply with the standard of a systematic review, for example, focusing more in detail on synthesis of the results and risk of bias. Further, firm conclusions about the role of anxiety as a risk factor of CVD awaits rigorous meta-analysis. As we only used English terms in our searches, any potential new findings published in a non-English language would not be discovered and included in our review.

## Conclusion and practical implications

In this paper, we have reviewed the current empirical status of anxiety as a risk factor for CVD independent of depression. It is evident that there still is substantial uncertainty about the status of anxiety as an independent risk marker for both incident and recurrent CVD. In our opinion, further research into this should take care to be population specific, measurement specific and outcome specific to elucidate this. Despite obvious limitations associated with narrative reviews, the results suggest that the current standing of anxiety as an independent risk marker of CVD is ‘possible’, and should not be treated interchangeable with depression, despite their co-morbidity. Hence, international guidelines for CVD prevention (e.g. Piepoli et al., 2016) should be revised accordingly pending sufficient empirical evidence and scrutinised investigation allowing for firm conclusions. Further, when targeting mental health to reduce the risk of CVD, treating depression should be prioritised.
